# Out-of-plane Stokes imaging polarimeter for early skin cancer diagnosis

**DOI:** 10.1117/1.JBO.17.7.076014

**Published:** 2012-07-09

**Authors:** Pejhman Ghassemi, Paul Lemaillet, Thomas A. Germer, Jeffrey W. Shupp, Suraj S. Venna, Marc E. Boisvert, Katherine E. Flanagan, Marion H. Jordan, Jessica C. Ramella-Roman

**Affiliations:** a Catholic University of America, Washington, District of Columbia 20064; b National Institute of Standards and Technology, Gaithersburg, Maryland 20899; c Washington Hospital Center, MedStar Health Research Institute, Washington, District of Columbia 20010

**Keywords:** Stokes imaging polarimeter, rough surface scattering, benign nevi, melanocytic nevus, melanoma

## Abstract

Optimal treatment of skin cancer before it metastasizes critically depends on early diagnosis and treatment. Imaging spectroscopy and polarized remittance have been utilized in the past for diagnostic purposes, but valuable information can be also obtained from the analysis of skin roughness. For this purpose, we have developed an out-of-plane hemispherical Stokes imaging polarimeter designed to monitor potential skin neoplasia based on a roughness assessment of the epidermis. The system was utilized to study the rough surface scattering for wax samples and human skin. The scattering by rough skin—simulating phantoms showed behavior that is reasonably described by a facet scattering model. Clinical tests were conducted on patients grouped as follows: benign nevi, melanocytic nevus, melanoma, and normal skin. Images were captured and analyzed, and polarization properties are presented in terms of the principal angle of the polarization ellipse and the degree of polarization. In the former case, there is separation between different groups of patients for some incidence azimuth angles. In the latter, separation between different skin samples for various incidence azimuth angles is observed.

## 1 Introduction

Melanoma is the most deadly form of skin cancer, with a 14% mortality rate and 60,000 new cases per year in the US.^1^ Successful clinical detection of melanoma ranges between 40% and 80%,^2^ and accuracy of diagnosis depends heavily on clinician expertise. Improved diagnostic techniques for screening of melanoma would have a great impact on patient care and survival. Several methodologies and instrumentations have been used in the past few years for this purpose. Swanson et al.^3^ utilized an imaging system to diagnose skin lesions using several morphological and physiological parameters. These parameters were derived from predictive models of light absorption and scattering by chromophores such as hemoglobin, keratin, and melanin at different epidermal and dermal depths. Wan and Applegate^4^ developed a high-resolution molecular imaging technique, based on a fusion of spectroscopy and optical coherence microscopy to provide a strong contrast between melanotic and amelanotic regions. Han et al.^5^ presented a near-infrared (NIR) fluorescence imaging system with particular utility for direct *in vivo* characterization of cutaneous melanin. Yaroslavsky et al.^6^ utilized a multispectral polarized light imaging technique to enhance the skin lesion margin. Tuchin and colleagues^7^^,^^8^ developed several methods to reduce the confounding effect of light scattering inside the biological tissue and blood, which allowed them to increase the quality of optical imaging, especially for cancer diagnostics. Polarized light imaging has been used in the past as a noninvasive method for evaluating borders of nonpigmented lesions.^9^^,^^10^ Jacques and colleagues^11^^,^^12^ used polarized light imaging to determine the margins of certain skin cancers by relying on the contrast provided by a cancer-induced disruption of the underlying collagen matrix. A similar effect is produced by scar tissue that exhibits a lower degree of polarization than normal tissue, possibly induced by the random restructuring of collagen.^13^ Ghosh and colleagues^14^^–^^16^ reported a sensitive polarimetric platform and presented a Mueller matrix decomposition methodology and its application to decouple the combined polarization information from tissue. Furthermore, Vitkin and colleagues^17^^,^^18^ and Ghosh and colleagues^19^^,^^20^ presented several studies on tissue polarimetry and its application in biomedical imaging and diagnosis.

Recently, several authors have been evaluating superficial structural components, such as roughness, as a way of discriminating melanocytic from normal pigmented lesions. For example, Tchvialeva and colleagues^21^^,^^22^ developed a methodology for quantifying skin surface roughness using laser speckle contrast. Pacheco et al.^23^ used microtopographic inspection of the skin surface to determine a unique pattern of roughness for benign and malignant skin lesions. Gareau et al.^24^ used reflectance confocal microscopy to introduce a roughness score for the dermal-epidermal junction.

Polarized backscattering measurements offer high sensitivity to many types of defects, including surface roughness, subsurface features, and particulate contaminants.^25^^–^^28^ However, it is often difficult to distinguish between these various scattering mechanisms. Germer and colleagues^25^^,^^29^ used light scattering ellipsometry to distinguish surface from subsurface scattering for a variety of inorganic materials, such as silicon wafers, glass, and metals. The authors found that different single-scattering mechanisms did not depolarize the light, but yielded different polarization states. Our group^29^ has applied similar techniques to the study of skin, demonstrating that rough-surface effects of skin could be minimized using out-of-plane polarized illumination and detection. Finally spectropolarimetric techniques have been used successfully to assess skin roughness including wrinkles.^10^^,^^30^

In this paper, we introduce a novel polarimetric system that captures the illumination-direction dependence of the polarization state of scattering from skin. After calibrating the Stokes polarimeteric imaging module, the polarization state of each light illumination part was aligned precisely with a set of gold roughness standards, and the overall system was tested with optical phantoms. A facet scattering model was used to validate the results of calibration. After validation of the method against roughness standards, we tested the instrument in a clinical study on human skin. Polarization parameters such as the principal angle of the polarization ellipse and the degree of polarization show meaningful behavior in relation to the change of illumination azimuth angle.

## 2 Theory

**Fig. 1:**
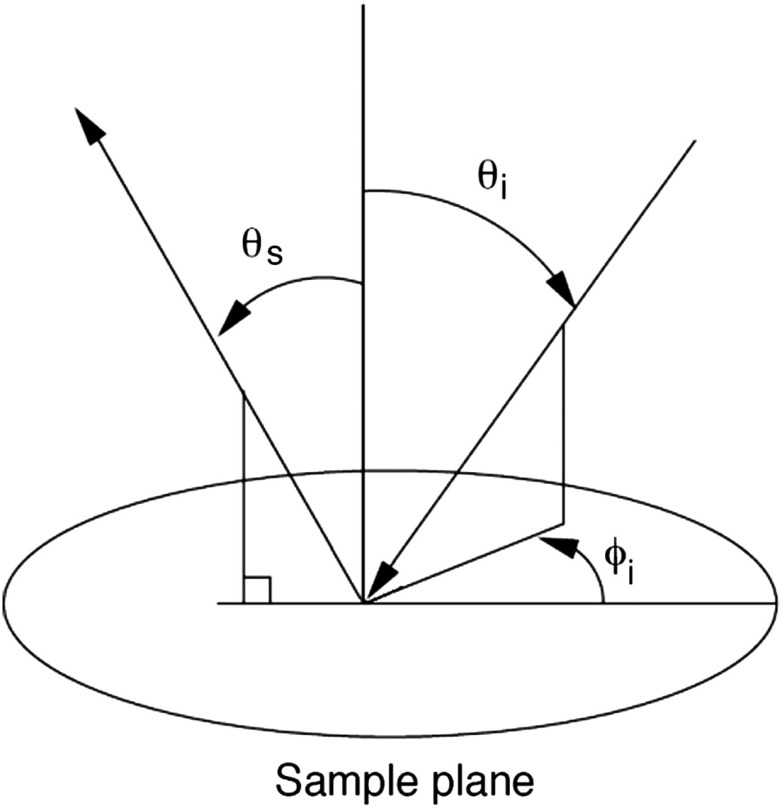
The geometry for out-of-plane scattering; $\theta_{i}$ is the incidence polar angle, $\theta_{s}$ is the scattered polar angle, and $\phi_{i}$ is the incidence azimuth angle.

The bidirectional reflectance distribution function (BRDF), $f_{r}$, is commonly used to describe scattering by surfaces.^31^ For isotropic materials, the BRDF is a function of the incident polar angle $\theta_{i}$, the incident azimuthal angle $\phi_{i}$, and the polar scattering angle $\theta_{s}$, and is given by

$$f_{r}=\underset{\Omega \to 0}{\lim}\frac{\Phi_{i}}{\Phi_{s}\Omega \cos\;\theta_{s,}},$$ (1)

where $\Phi_{s}$ is the scattered light power, $\Phi_{i}$ is the incident light power, and Ω is the collection solid angle (see Fig. 1). The BRDF is a function of the incident light polarization and, if expressed as a Mueller matrix, can include information about the outgoing light polarization.

Skin is a very complex layered medium exhibiting multiple scattering components. To simulate such complexity, we would be expected to include a combination of numerous scattering mechanisms, which include rough-surface scattering from the stratum corneum and dermoepidermal junction, single scattering from cell nuclei, and multiple scattering from cells and collagen bundles. Significant insight in light scattering, however, can be obtained from a few simple models. Subsurface scattering has been modeled in the past as a sum of a single scattering component, based on a Henyey-Greenstein phase function, and a diffuse highly scattering component.^32^

In this paper, we chose to treat the scattering with a rough surface model for the air/stratum corneum interface and a totally diffuse, depolarizing model for the volume scattering beneath the surface. The facet scattering model for rough surfaces makes the assumption that the features on the surface and the correlation lengths are large compared to the wavelength and that the surface can be represented by random flat facets, each of which specularly reflects light according to its orientation. The BRDF is then given by^33^

$$f_{r}=\frac{P(\varsigma_{x},\varsigma_{y})}{4\cos\;\theta_{i}\cos\;\theta_{s}\cos^{4}\theta_{n}}R,$$ (2)

where $\theta_{i}$ is the incident angle, $\theta_{s}$ is the reflected, scattered angle, $\theta_{n}$ is the angle of the facet normal to the mean surface normal, and $P(\varsigma_{x},\varsigma_{y})$ is the slope distribution function describing the facet orientations, where $\varsigma_{x}$ and $\varsigma_{y}$ are the facet slopes in the x and y directions, respectively. The reflectance of the facets, R, is presumed to be given by the Fresnel equations and is the only contribution to the polarimetric behavior of the BRDF. That is, the slope distribution function and the polarization are independent. Roughness may also be treated using first-order vector perturbation theory;^34^ however, the roughness of skin is generally considered to be too large for perturbation theory to be applicable. The theory does share the behavior that the light-scattering polarization is independent of the roughness statistics.

Because we are treating the volume scattering as completely depolarizing, the total scattering signal has a polarized part that indicates the rough-surface scattering and a depolarizing part that carries little information. If the scattered Stokes vector is S, we can uniquely decompose it into its polarized component, $S^{\mathrm{pol}}$, and its unpolarized component, $S^{\mathrm{unpol}}$,

$$S=S^{\mathrm{Pol}}+S^{\mathrm{UnPol}}=(\begin{array}{c} \sqrt{S_{1}^{2}+S_{2}^{2}+S_{3}^{2}}\\ S_{1}\\ S_{2}\\ S_{3} \end{array})+(\begin{array}{c} S_{0}-\sqrt{S_{1}^{2}+S_{2}^{2}+S_{3}^{2}}\\ 0\\ 0\\ 0 \end{array}).$$ (3)

We thus characterize the scattering polarization by the principal angle of the polarization ellipse, η, and the degree of polarization, DOP, or

$$\eta =\frac{1}{2}\arctan(\frac{S_{2}}{S_{1}})\;\mathrm{DOP}=\frac{\sqrt{S_{1}^{2}+S_{2}^{2}+S_{3}^{2}}}{S_{0}}.$$ (4)

We use the Modeled Integrated Scatter Tool (MIST) to evaluate the facet scattering model.^35^ The MIST program is designed to evaluate the reflectance integrated over a solid angle Ω,

$$\rho (\Omega )=\int_{\Omega }f_{r}\;\cos\;\theta_{s}d\Omega ,$$ (5)

for a wide variety of scattering models. The program can evaluate the integrated reflectance as a function of model parameters (e.g., index of refraction and slope distribution function), geometric parameters (e.g., incident direction and collection geometry), wavelength, and polarization.

Out-of-plane scattering measurement has been shown to be helpful for distinguishing between different scattering mechanisms.^25^^,^^27^ In the plane of incidence, any isotropic material will not mix polarization (defined by the electric field) parallel to the plane (p-polarization) with that perpendicular to the plane (s-polarization). Furthermore, the polarization of light scattered by many models—including the facet model, subsurface Rayleigh models, and models for particles above the surface—show very little polarimetric differentiation for s-polarized incident light. As a result, the greatest polarimetric differentiation between scattering sources occurs when viewing the samples out of the plane of incidence with the p-polarized incident light. However, when illuminating the samples from many directions, adequate differentiation can be obtained when the incident polarization is linearly polarized at 45 deg for all incident directions.

## 3 Materials and Methods

### 3.1 Experimental Setup

**Fig. 2:**
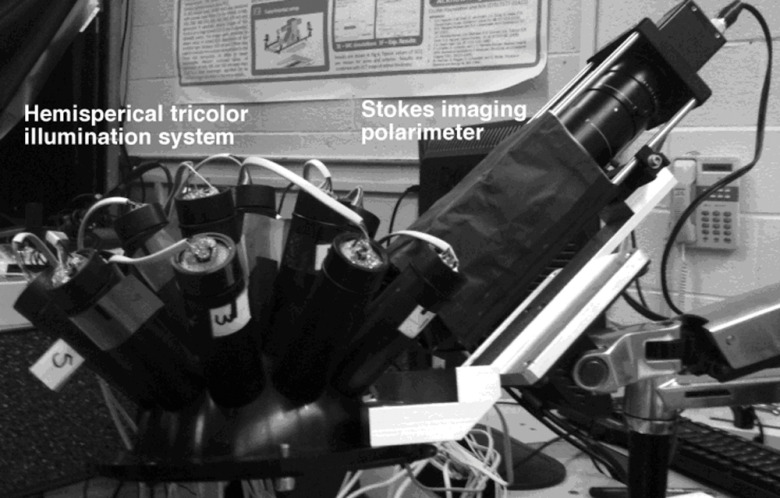
The hemispherical spectropolarimetric scattering instrument.

Our hemispherical Stokes imaging system is designed to enable multi-angle out-of-plane measurements without any moving parts. This system is composed of a Stokes imaging polarimeter with a 12-bit digital charge-coupled device (CCD) black and white camera (Dalsa Genie, Billerica, MA) positioned at one scattering angle $\theta_{s}=49\;\deg$ as shown in Fig. 2.* Sixteen illumination tubes are distributed about a hemisphere. Illuminators 1 to 9 are centered at $\theta_{i}=49\;\deg$, illuminators 10 to 15 are centered $\theta_{i}=24\;\deg$, and illuminator 16 is centered on the surface normal, $\theta_{i}=0\;\deg$. The choice of polar incidence angles (0 deg, 24 deg, and 49 deg) is made simply by attempting to cover the hemisphere with ports. Each illumination tube contains a tricolor light emitting diode (LED) followed by a polarizer $P_{1}$ (Edmund Optics, Barrington, NJ), and a lens $1_{1}$(Edmund Optics, Barrington, NJ). The tricolor LED emits in three bands: red, centered on λ=630  nm; green, centered on λ=525  nm; and blue, centered on λ=472  nm (widths=30  nm measured as the full width at half maximum). Each LED is controlled with a digital-to-analog module (Measurement Computing Corp., Norton, MA). Each tube illuminates the sample, located at the center of the hemisphere, with an approximately 2-cm-diameter beam.

**Fig. 3:**
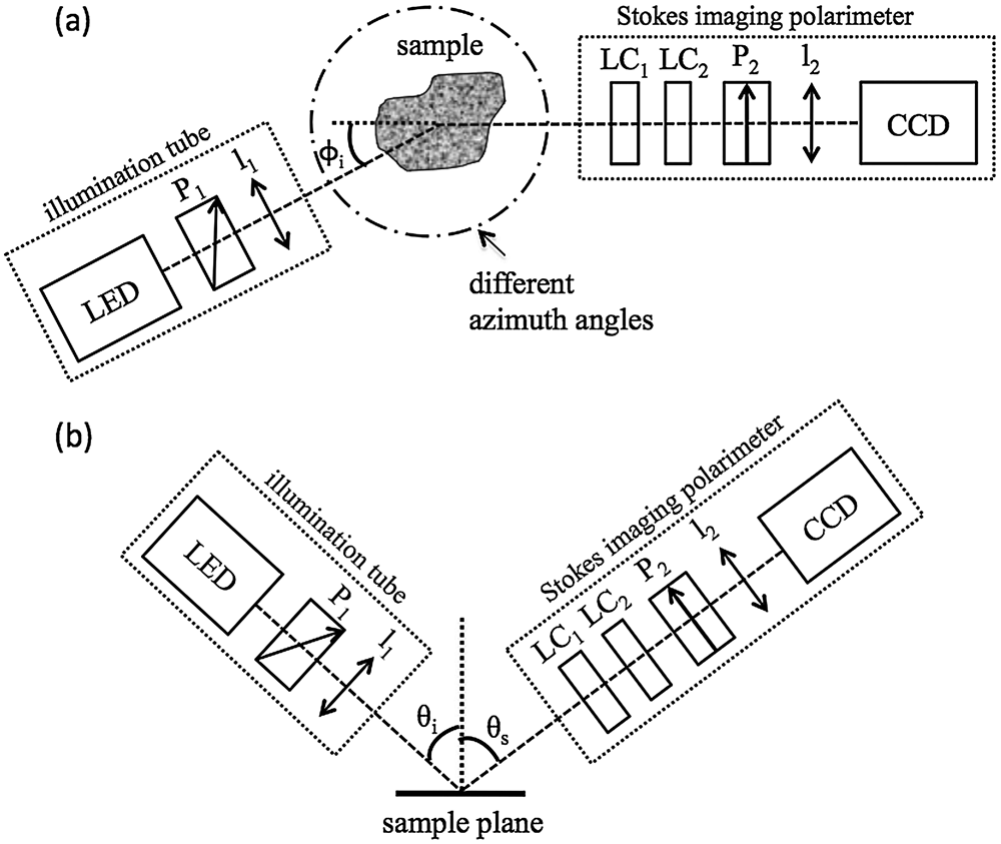
System geometry showing one illumination tube at incidence angle of $\theta_{i}$ and azimuth angle of $\phi_{i}$ and the Stokes imaging polarimeter at scattered angle of $\theta_{s}$; top view (a) and side view (b). LED is a three-color light source, $P_{1}$ is the illumination polarizer at 45 deg with respect to the plane of incidence, $l_{1}$ is the collimating lens, ${\mathrm{LC}}_{1}$ and ${\mathrm{LC}}_{2}$ are liquid crystal retarders, $P_{2}$ is the vertical reference polarizer, and $l_{2}$ is a zoom lens. CCD is a black-and-white camera used for acquisition.

The polarization state analyzer (PSA) consists of two nematic liquid crystal (${\mathrm{LC}}_{1}$ and ${\mathrm{LC}}_{2}$) variable retarders (Meadowlark Optics Inc., Frederick, CO) followed by a vertical linear dichroic polarizer, $P_{2}$. Figure 3 presents the system geometry, showing one illumination tube and the Stokes imaging polarimeter. Images are captured by the fast-acquisition CCD camera attached to a zoom lens $l_{2}$ (Computar, Commack, NY). The LC cells are mounted on manual rotation stages (precision of 1 deg) that help adjust their fast-axis rotation angles, $\gamma_{1}$ and $\gamma_{2}$, with respect to the axis of the polarizer. It takes 2 min to obtain a set of Stokes vector images for each of the 16 illumination directions using this setup.

### 3.2 Calibration of the Stokes Polarimeter

The calibration of the Stokes imaging polarimeter followed a method outlined by Boulbry et al.^36^ that involved generating a set of known Stokes vectors and computing the data reduction matrix W from the measured intensities and the calibration Stokes vectors. A Stokes vector, which describes the polarization state of light propagating in a particular direction, takes the form

$$S=(\begin{array}{c} I_{x}+I_{y}\\ I_{x}-I_{y}\\ I_{45^{{}^{\circ}}}-I_{-45^{{}^{\circ}}}\\ I_{\mathrm{rcp}}-I_{\mathrm{lcp}} \end{array}),$$ (6)

where the first term, $S_{1}$, is the total intensity, and $I_{j}$ is the intensity at various states of polarization with j=(x,y,45  deg,±45   deg,rcp,lcp). The x (y) direction is defined in our measurements as the direction parallel (perpendicular) to the plane defined by the viewing direction and the surface normal. The acronyms rcp and lcp stand for right-circular and left-circular polarization, respectively, whereas ±45  deg stands for linear polarization of light at ±45  deg about the normal. One cannot measure the elements of S directly and thus must compute them through polarization analysis measurements. The relationship between the Stokes elements and the measured intensities can be expressed in matrix form as

S=WI, (7)

where W is the data reduction matrix and I is a vector of the measured intensities for different combinations of retardances for ${\mathrm{LC}}_{1}$ and ${\mathrm{LC}}_{2}$. A minimum of four intensity measurements is required to compute S.^12^ Calibrating the polarimeter means assessing W.

The LC azimuth angles as well as their driving voltages were chosen to minimize the condition number of W, Cond(W) (defined as the ratio of the largest to the smallest of the singular values of W).^37^^,^^38^ The higher the condition number, the less linearly independent are the columns and rows of W. Minimizing the condition number maximizes the relative importance of each of measurements, increasing system stability and decreasing noise propagation.^36^^,^^39^ We computed a single set of driving voltages (hence, retardances) and angles for LC1 and LC2 that minimized the sum of squares of the condition number for each of the three available illumination wavelengths. The LC cells were modeled as linear retarders in the simulations. The retardation curves as a functions of the driving voltage were provided by the supplier at λ=630  nm and were estimated for 472 and 525 nm based on the assumption of negligible dispersion. Hence, the chosen azimuth angles are $\gamma_{1}=22\;\deg$ and $\gamma_{2}=45\;\deg$, and the retardances for the four measurements $\left(\delta_{1},\delta_{2})=({\delta_{1}}^{a},{\delta_{2}}^{a}\right)$, (${\delta_{1}}^{a},{\delta_{2}}^{b}$), (${\delta_{1}}^{b},{\delta_{2}}^{a}$), (${\delta_{1}}^{b},{\delta_{2}}^{b}$), along with the corresponding condition numbers, are presented in Table 1. Since we chose to use the same set of driving voltages for each of the three illumination wavelengths, these parameters led to condition numbers for W that are not the ideal minimum of 1.73 reported by Tyo.^39^

**Table 1:** Retardance angles used on the LC retarders.

Retardance	λ=472 nm (deg)	λ=525 nm (deg)	λ=630 nm (deg)
${\delta_{1}}^{a}$	173.2	208.8	232.3
${\delta_{2}}^{a}$	119.3	143.8	160
${\delta_{1}}^{b}$	20.9	25.2	28.1
${\delta_{2}}^{b}$	32	38.6	43
Cond (W)	2.18	1.81	2.34

To calibrate the polarimeter, we set up a polarization state generator right in front of the imaging arm to generate an input set of known Stokes vectors. These consisted of a set of 18 linearly polarized states generated by a rotating linear polarizer, and 18 circularly polarized states generated by a rotating linear polarizer positioned before a quasi-achromatic quarter-wave plate. The vectors spanned the equator and a meridian of the Poincaré sphere. The original calibration procedure by Boulbry et al.^36^ used a Fresnel rhomb instead of an achromatic quarter-wave plate, which required a beam displacement that made the calibration harder. We chose a quasi-achromatic quarter-wave plate to prevent the beam displacement at the expense of the achromaticity and accuracy of the retardance. However, the resulting calibration of the polarimeter is less than 3%, which is a typical value for this type of measurement.^40^

For each illumination tube, the orientation of the polarizer was set to 45 deg from the incident plane, i.e., the plane defined by the sample normal and the illumination direction. Because the setup is not built on a goniometric platform, it is somewhat difficult to adjust the orientation of the linear polarizer in each illumination tube. To aid in the adjustment of the incident polarizer orientations, we measured the Stokes vector for λ=630  nm illumination from each of the tubes and that of four reference aluminum samples that were roughened by electrodischarge machining at different levels of roughness and coated with gold.^30^ A detailed process of fine adjustment of each illumination polarizer is available in our previous reported study.^10^ We calculated ellipticity and the principal angle of polarization of the measured Stokes vectors. The data were then modeled using a facet scattering model from the SCATMECH/MIST library.^35^ There is good agreement between the results of the model and experimental measurements of the gold samples obtained using Stokes polarimeter with well-aligned illumination polarizers.

### 3.3 Phantom Samples

The system was tested using skin-simulating phantoms. We built surface-roughened optical phantoms that mimic skin’s optical properties. The values provided for human dermis available in the literature^41^ are, at λ=630  nm, the absorption coefficient, $\mu_{a}$, which varies from 0.1 to $0.2\;{\mathrm{mm}}^{-1}$, and the reduced scattering coefficient, $\mu_{s}^{\prime }$, which varies from 3.55 to $5\;{\mathrm{mm}}^{-1}$. We used wax (Batik Wax, Jacquard, Healdsburg, CA) as the casting material, since it is easy to mold; however, wax has scattering properties that needed to be accounted for in the final result.

The phantoms were made as follows. Wax was melted on a hotplate stirrer, and ${\mathrm{TiO}}_{2}$ was added to adjust $\mu_{s}^{\prime }$. The mixture was stirred until it was visibly homogeneous, and black wax (Jacquard, Healdsburg, CA) was incorporated to adjust $\mu_{a}$. This preparation was poured into two molds. One had a rough imprint (based on sandpaper with ANSI grade 60) at the bottom that created a 5-mm-thick phantom for polarization measurements. The other provided a smooth 2-mm-thick phantom that was used to measure the bulk optical properties. The inverse adding doubling (IAD) program^42^ was used with measurements of the total reflectance and the total transmittance to compute $\mu_{s}^{\prime }$ and $\mu_{a}$. These measurements were obtained with an integrating sphere (Labsphere, 10.2 cm diameter, calibrated wall reflectance of 97.1%) and a He-Ne laser source λ=632.8  nm (CVI Melles Griot, Albuquerque, NM). Table 2 presents the measured optical parameters of the four smooth wax phantoms. The anisotropy factor for ${\mathrm{TiO}}_{2}$ has been reported as equal to 0.5 by several authors at the wavelength of interest.^43^^,^^44^ All phantoms exhibited optical properties that fit the reference human dermis values, except phantom B.

**Table 2:** Optical properties of the wax phantoms.

Phantom	$\mu_{a}({\mathrm{mm}}^{-1})$		$\mu_{s}^{\prime }({\mathrm{mm}}^{-1})$	
	Mean	Standard deviation	Mean	Standard deviation
A	0.127	0.0027	3.749	0.0039
B	0.142	0.0054	6.897	0.2567
C	0.204	0.0047	4.005	0.1935
D	0.216	0.0099	4.770	0.2762

**Fig. 4:**
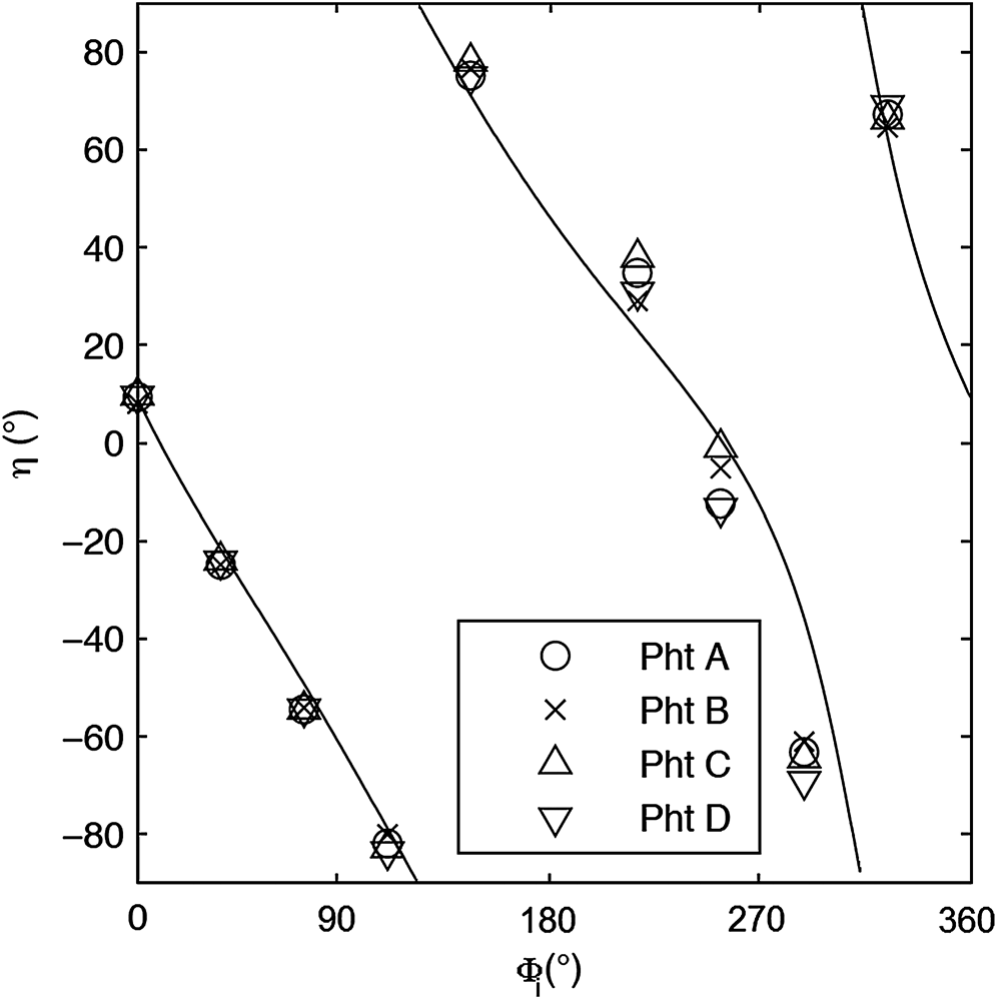
The principal angle of polarization, h, for a set of wax samples illuminated sequentially by tubes 1 to 9. The curve denotes the facet scattering model (n=1.42 and k=0, at l=630  nm).

**Fig. 5:**
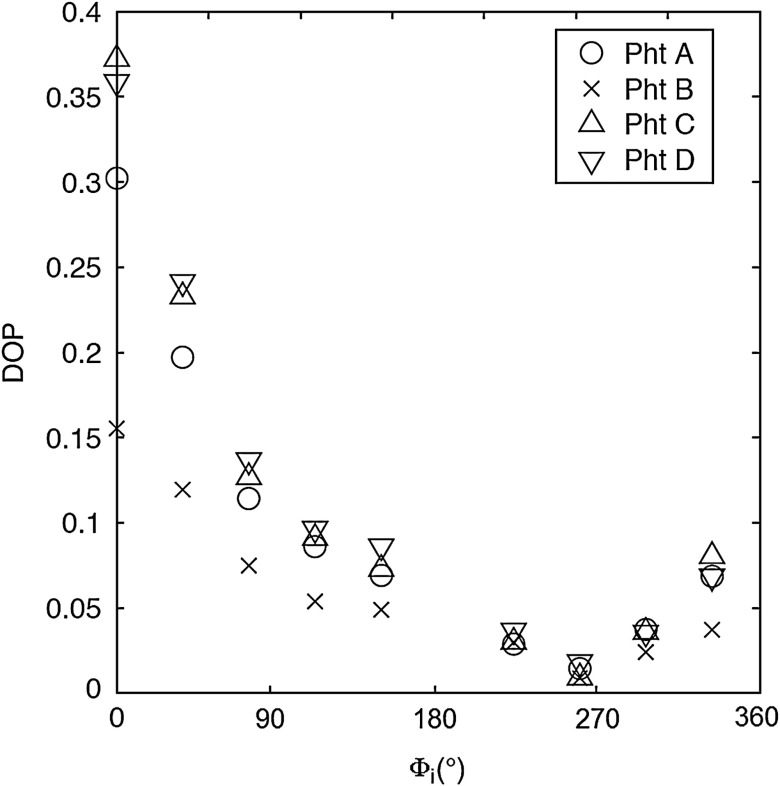
Degree of polarization for a set of wax samples illuminated sequentially by tubes 1 to 9.

For each phantom, we computed the Stokes vector versus the illumination azimuth direction, $\phi_{i}$, following the procedure previously explained. We used only one illumination wavelength, λ=630  nm. The principal angle of the polarization ellipse, η, and the degree of polarization, DOP, were estimated as functions of the illumination azimuth angle and were compared to the same parameters predicted by the facet scattering model (with optical constants n=1.42 and k=0 for wax at $\lambda =630^{{}^{\circ}}\;\mathrm{nm}$), shown for tubes 1 to 9 in Fig. 4 (η) and Fig. 5 (DOP). The principal angle of polarization shows a cyclical behavior as a function of the illumination azimuth angle. The incoming polarization is linearly polarized at 45 deg to the local incident plane for each illumination direction. Therefore the resulting principal angle of the polarization is not symmetrical. Furthermore the discontinuities visible in the graph are due to the mathematical formulation of the principal angle of polarization, where a small change in $S_{1}$ or $S_{2}$ can cause shift from −90 to 90 deg as seen in Fig. 4. Figures 4 and 5 show that although the scattering and absorption coefficients do not affect the resulting principal angle of polarization, they do influence the degree of polarization. That is, the phantom with the highest scattering coefficient (phantom B) consistently has the lowest degree of polarization.

### 3.4 Skin Samples

**Fig. 6:**
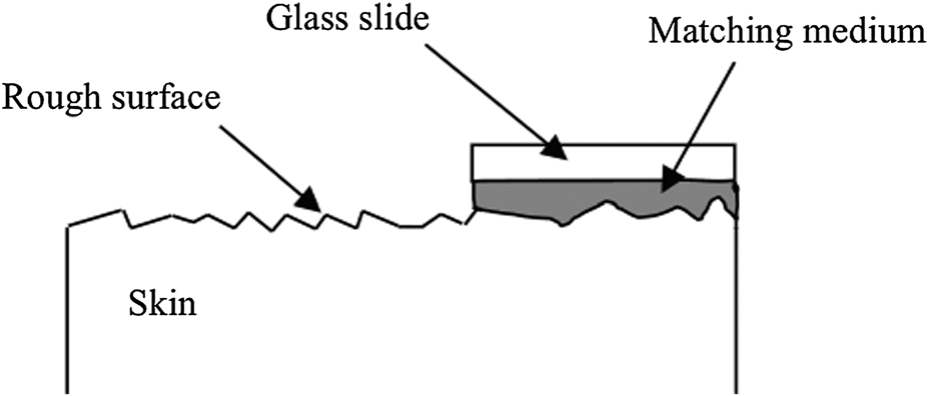
Portions of the skin were covered with a glass slide and matching fluid.

To assess different rough-surface scattering effects due to multiple sources of skin scattering, polarization studies were conducted on Caucasian skin *in vivo*. A portion of the skin was smeared with index-matching gel and covered with a thin glass slide, as illustrated in Fig. 6. A glass slide and an index-matching fluid (mineral oil) were used to minimize the effect of skin roughness and to increase the sensitivity of the measurement to subsurface features. The covered portion allowed for a quick elimination of the rough-surface effect in one section of the image. The birefringence of the glass slide and the matching fluid were insignificant; therefore, they were not expected to alter the polarization state. The remaining part of the skin sample was left untouched; rough-surface scattering effects were most visible in this section. The MIST program was utilized to evaluate the facet scattering model for each experiment, with average index of refraction n=1.38 for skin tissue^45^ at λ=630  nm and 45 deg linearly polarized illumination based on the geometric parameter of the imaging system.

**Fig. 7:**
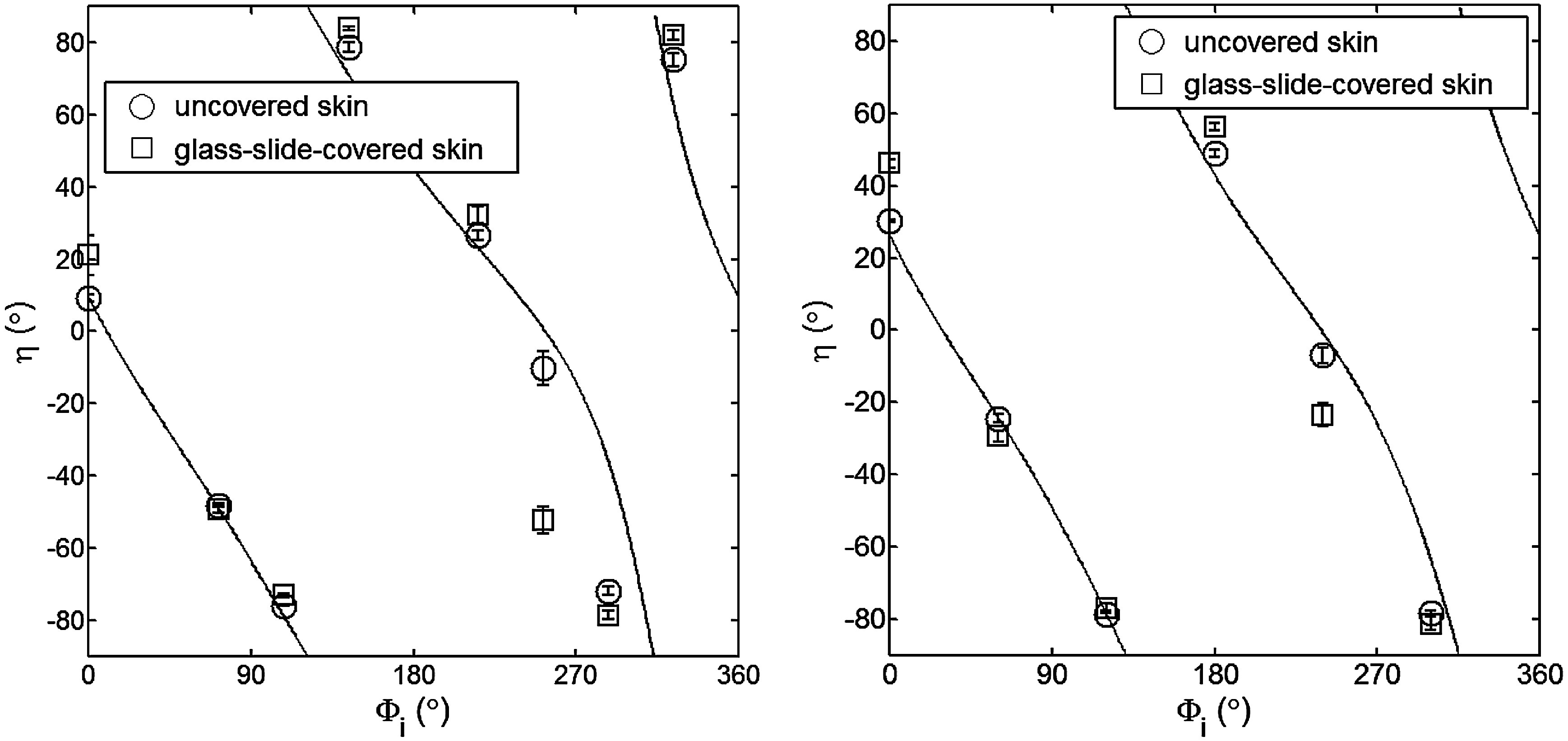
Principal angle of polarization for a glass-slide-covered and uncovered skin sample for (a) $q_{i}=49\;\deg$ (illumination tubes 1 to 9), and (b) $q_{i}=24\;\deg$ (illumination tubes 10 to 15). The curves represent the facet model.

Figure 7 shows results for the glass-slide-covered and uncovered portions of the skin sample for two different incidence angles $\theta_{i}$. The graphs show the $\phi_{i}$-dependent variation of the principal angle of polarization η for $\theta_{i}=24\;\deg$ and $\theta_{i}=49\;\deg$. The error bars in all plots represent the standard deviation of the mean of the data for four samples. Results of uncovered skin mostly follow the facet scattering model. There is a marked shift toward the predictions of the facet scattering model for the glass-slide-covered skin. These results highlight the effect of rough-surface scattering mechanisms in this analysis.

## 4 Results

**Fig. 8:**
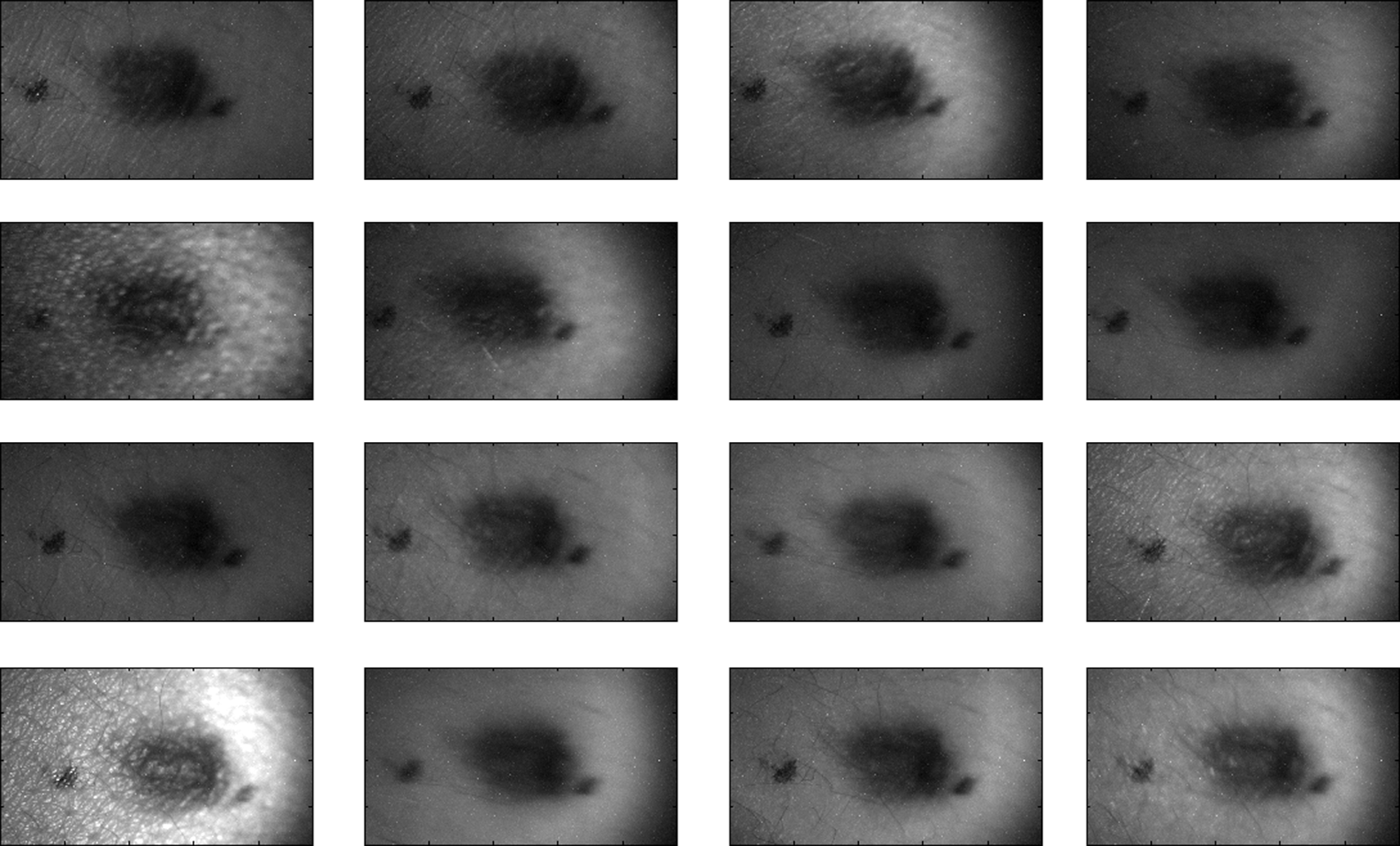
Sequential intensity images at the 16 different illumination azimuth angles captured by the Stokes polarimeter from a 9-×7-  mm benign nevi located at midline mid-back at T5 of a 26-year-old Caucasian female. First row shows illumination tubes 1 to 4, second row 5 to 8, third row 9 to 12, and fourth row 13 to 16.

An ongoing clinical trial is being conducted at the Washington Cancer Institute’s Melanoma Center, and Institutional Review Board approval and informed consent have been obtained. The goal of the study is to assess the validity of rough-surface scattering as a diagnostic tool for melanoma. A total of 13 individuals have been imaged so far. All volunteers were Caucasian with fair skin (types I and II in the Fitzpatrick scale^46^). Nine benign pigmented nevi, two melanocytic nevus, two melanoma, and 13 normal skin tissue were imaged. All suspicious lesions were excised and sent to pathology for evaluation. Sequential azimuthal illumination images of a benign nevus from one patient are depicted in Fig. 8.

**Fig. 9:**
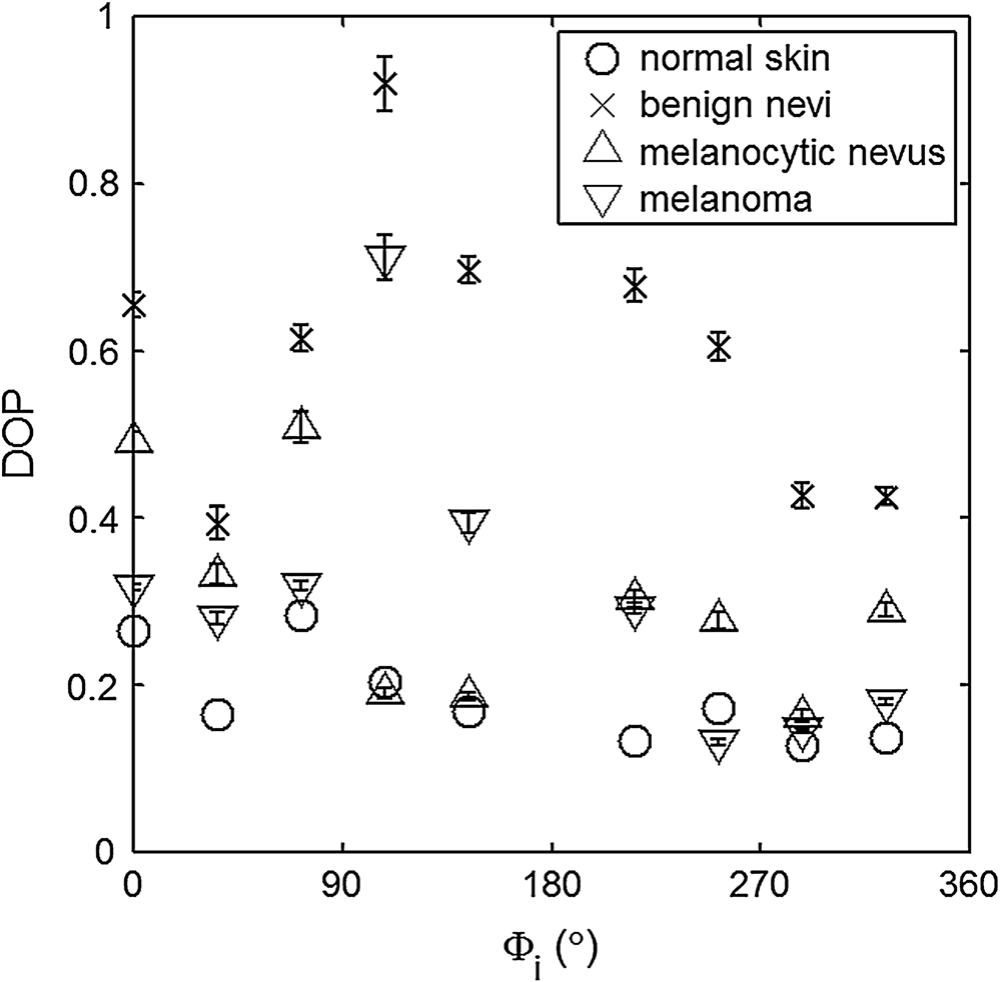
Degree of polarization for skin illuminated sequentially by tubes 1 to 9 ($q_{i}=49\;\deg$). Circles are averaged normal skin values (Caucasian), whereas crosses are benign nevi, upright triangles are melanocytic nevus, and upside-down triangles are melanoma.

**Fig. 10:**
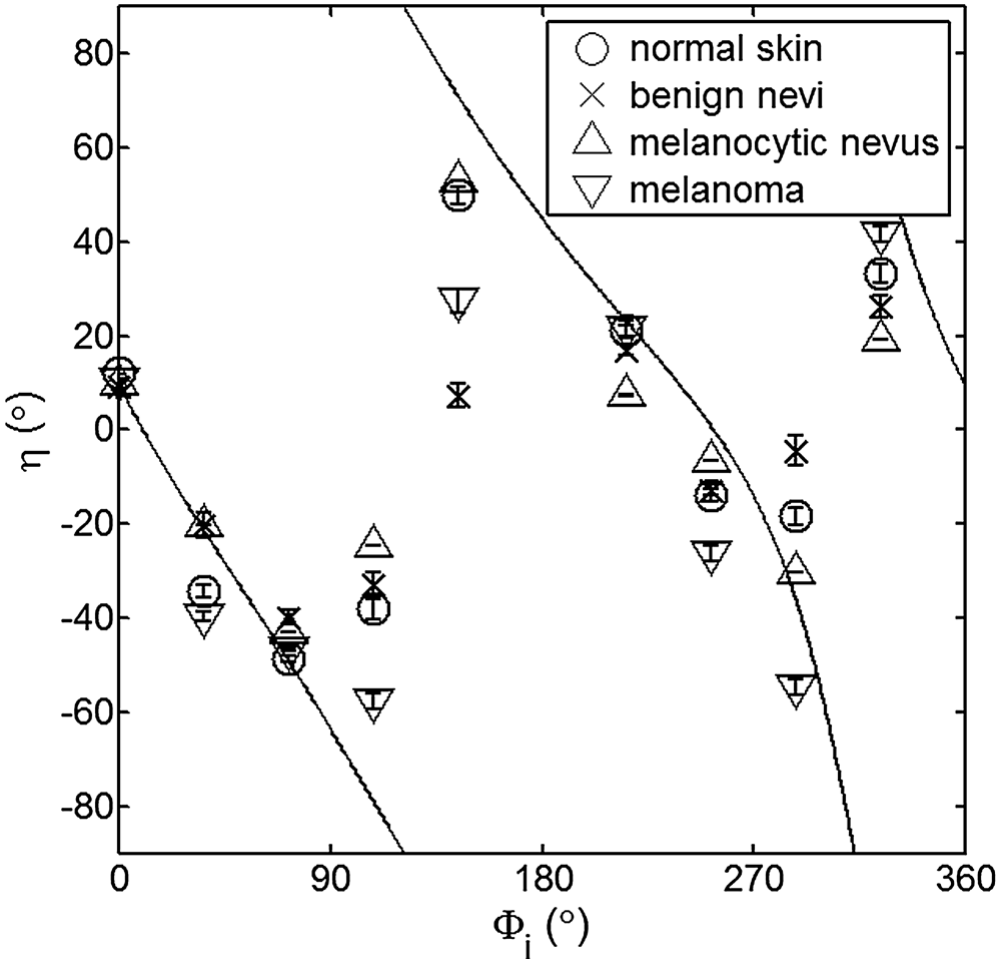
Principal angle of polarization for skin illuminated sequentially by tubes 1 to 9 ($\theta_{i}=49\;\deg$). Filled circles are averaged normal skin values (Caucasian), whereas crosses are benign nevi, upright triangles are melanocytic nevus, and upside-down triangles are melanoma. The curve represents the predictions of the facet model described in the text.

**Fig. 11:**
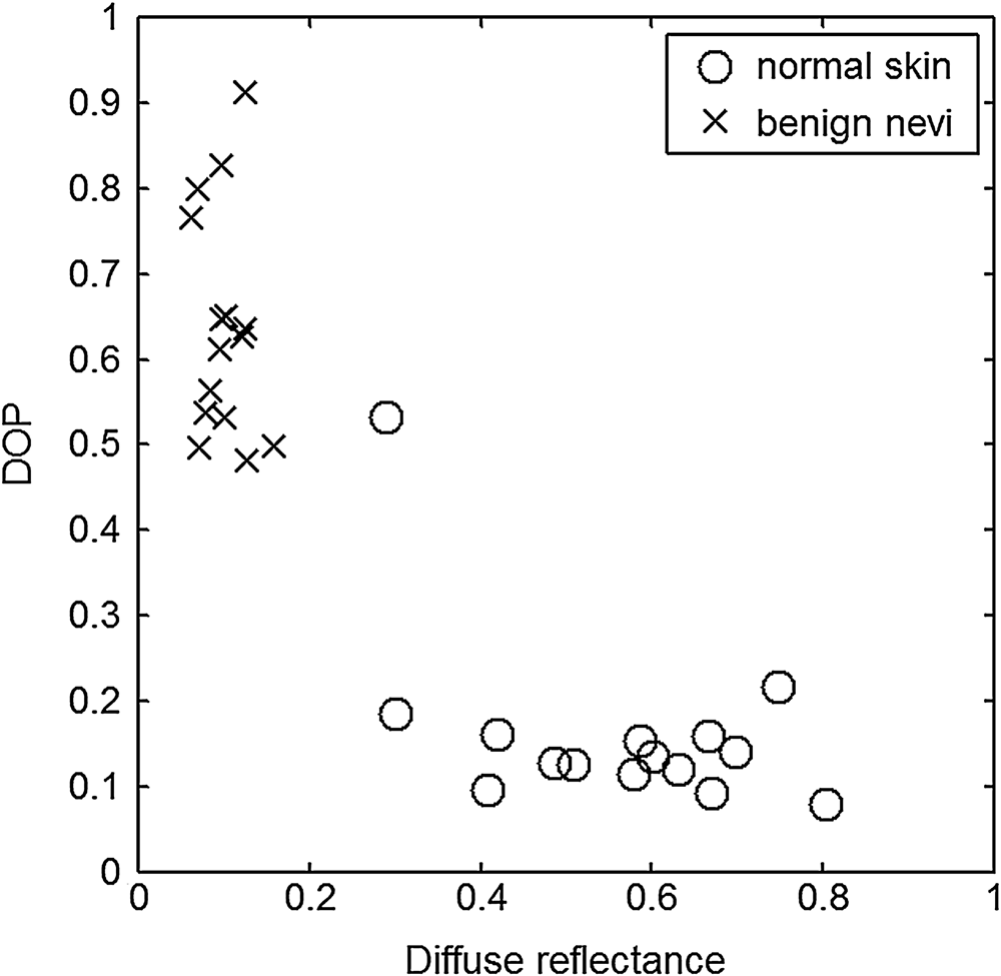
Degree of polarization versus diffuse reflectance measured at various incidence azimuth angles of a benign nevus (crosses) located at right hip lower abdomen, and its normal surrounding tissue (circles) from a 29-year-old Caucasian female.

The principal angle of polarization and the degree of polarization were calculated for each nevus or lesion and its surrounding area. The combined results are presented in Fig. 9 (for DOP) and Fig. 10 (for η), and in the former figure is compared to model results. The error bars in all plots are standard deviation of the mean for each group of patients; this is very low for DOP of normal skin and are therefore not depicted in Fig. 9. Maximum values of standard deviation for different groups of patients were benign nevi 0.02 for DOP and 4.30 deg for η; melanocytic nevus 0.03 for DOP and 5.20 deg for η; and melanoma 0.03 for DOP and 4.24 deg for η. For most of the azimuth incidence angles (except 216 and 282 deg), a separation exists between the three groups of pigmented lesions when observing the degree of polarization. In contrast, separation of patient groups is visible for only some of the azimuth incidence angles using the principal angle of polarization. Pigmented lesions have a higher absorption coefficient compared to normal skin. Therefore, the degree of polarization of backscattered light for pigmented lesions is higher than that of normal skin tissue as expected due to the Umov effect^47^ and the loss of highly scattered light in the overall diffuse reflectance from pigmented lesions.^48^ The relationship between diffuse reflectance from the skin tissue and degree of polarization, DOP, is explored in Fig. 11. Furthermore, scattering from surface and subsurface structures of melanocytic nevi and melanoma is higher (possibly due to greater roughness) compared to benign nevi (less roughness). The degree of polarization of backscattered light from the melanocytic nevi and melanoma should thus be lower than that of the benign lesion.

## 5 Discussion

We have shown that out-of-plane Stokes imaging polarimetry can provide information regarding rough-surface scattering, including that from highly scattering and absorbing tissue. The system was calibrated by generating a set of known Stokes vectors and computing a data reduction matrix using a previously published calibration methodology. The measurements rely on out-of-plane polarized illumination with polarization-sensitive viewing. The system was utilized to study rough-surface scattering from wax samples and human skin. The metrics utilized for analysis are the principal angle of polarization ellipse and the degree of polarization. Based on these, the scattering by rough skin—simulating phantoms exhibited behavior that is reasonably described by a facet scattering model.

The ultimate goal of this study was to demonstrate that a melanoma lesion has a different superficial structure (roughness) than a normal pigmented lesion. A few studies, mostly based on speckle sensing, have pointed to this phenomenon, although the biological mechanisms are not clear at present. One hypothesis is that early-stage melanocytic cells form nodes at the dermal—epidermal junction. At later stages, melanomas progress to the radial growth phase and vertical growth phase and consequently invade dermal component, changing its architecture.^49^ This change is reflected at the surface and could be responsible for the different roughness. Using the out-of-plane imaging system, we believe information about roughness structure of different groups of patients can be gathered. We applied our methodology to measure roughness properties between four groups of patients including normal skin, benign nevi, melanocytic nevus, and melanoma. Although our results are very preliminary due to the small population, we note a separation between the degrees of polarization of these groups. Particularly in Fig. 9, representing the degree of polarization for four populations, the melanoma data seem to be separated from the other data at all angles except two (216 deg and 282 deg). The degree of polarization, though, is influenced not only by roughness but also by the media scattering and absorbing properties, as we have shown in our wax phantoms study.

The more interesting metric to us is the principal angle of polarization, which is influenced primarily by different rough-surface scattering mechanisms. Again, looking at the wax study, we note that all the rough phantoms have similar rough structure, therefore they all present the same η behavior regardless of their scattering properties. Only when the rough surface structure is changed, as in our experiment with human skin covered with a glass slide, do we note a different η behavior, since the mechanisms of rough-surface scatter are completely different, although that is true only for few azimuth angles (0 deg and 252 deg at $\theta_{i}=49\;\deg$; 0 and 240 deg at $\theta_{i}=24\;\deg$). Similarly, in the human data, the principle angle of polarization for melanoma deviates from a model behavior, melanocytic nevus, and benign nevi at few angles (36 deg, 108 deg, 144 deg, 252 deg, 282 deg, 324 deg). We believe this could be due to difference in the roughness of this particular lesion compared to normal skin or benign lesions. To truly generalize these preliminary findings, further studies are necessary to confirm our hypothesis. A clinical trial at the Washington Cancer Institute’s Melanoma Center is ongoing and will be the focus of future analysis.

## Footnotes

* Certain commercial equipment, instruments, or materials are identified in this paper in order to specify the experimental procedure adequately. Such identification is not intended to imply recommendation or endorsement by the National Institute of Standards and Technology, The Catholic University of America, or the Washington Hospital Center, nor is it intended to imply that the materials or equipment identified are necessarily the best available for the purpose.
